# Findings from a feasibility study of estradiol for hypogonadal women with cystic fibrosis-related bone disease

**DOI:** 10.1186/s40814-021-00897-x

**Published:** 2021-08-19

**Authors:** Malinda Wu, Rabindra Tirouvanziam, Neha Arora, Vin Tangpricha

**Affiliations:** 1 Division of Endocrinology, Department of Pediatrics, Emory University School of Medicine, Atlanta, GA USA; 2 Division of Pulmonology, Allergy, Cystic Fibrosis and Sleep, Department of Pediatrics, Center for CF and Airways Disease Research, Emory University School of Medicine, Atlanta, GA USA; 3 Emory University College of Arts and Sciences, Atlanta, GA USA; 4 Division of Endocrinology, Metabolism, and Lipids, Department of Medicine, Emory University School of Medicine, Atlanta, GA USA; 5 Atlanta VA Medical Center, Decatur, GA USA

**Keywords:** Cystic fibrosis, Osteoporosis, Cystic fibrosis-related bone disease, Estrogen, Estradiol, Hypogonadism, Young adult, Pre-menopausal

## Abstract

**Background:**

Advancements in therapies for patients with cystic fibrosis (CF) have decreased mortality, leading to increased prevalence of chronic complications including bone disease. CF-related bone disease (CFBD) is characterized by low bone mineral density (BMD) and fragility fractures. Estrogen deficiency increases bone resorption, resulting in decreased BMD that can be restored with estrogen replacement. Current CF guidelines recommend treating female hypogonadal patients with CFBD with estrogen replacement, but no prospective study has investigated the effects of estrogen supplementation on CFBD. Estrogen is known to modulate inflammatory markers and autoimmune diseases.

We proposed to test the hypothesis that estrogen status plays a critical role in optimizing bone health, modulating inflammation, preserving lung function, and maximizing quality of life in premenopausal women with CF.

**Methods:**

We planned a randomized, placebo-controlled, investigator- and patient-blinded, pilot trial with two parallel arms. Eligible subjects were women with CF 18–50 years old with hypogonadism and low BMD who were not taking systemic glucocorticoids, had not had a prior transplant, and did not have contraindications to oral estradiol. Subjects would be block randomized to receive oral estradiol or placebo for 6 months. The primary outcome was feasibility metrics. Secondary outcomes included relative changes in estradiol, bone turnover markers, lung function, inflammatory markers, and quality of life metrics. The study was funded through departmental funds.

**Results:**

Of 233 subjects screened, 86 subjects were women with CF 18–50 years old and none were eligible for participation. Most subjects were excluded due to absent DXA report (24%), normal BMD (22%), or use of systemic estrogen (16%). Due to difficulty recruiting the planned 52 subjects, the trial was closed for recruitment and no subjects were randomized.

**Conclusion:**

This study was designed to investigate the feasibility of a safety and efficacy trial of estrogen therapy for women with CF. Unfortunately, due to eligibility criteria, the study was unable to recruit subjects. This feasibility study highlights the need for improved BMD screening in young women with CF. Future study designs may require the incorporation of a screening DXA as part of subject recruitment.

**Trial registration:**

The study was registered on ClinicalTrials.gov (NCT03724955).

## Key messages regarding feasibility

1. What uncertainties existed regarding the feasibility?

A study to recruit and identify hypogonadal women with cystic fibrosis (CF) with low bone mineral density (BMD) had not been performed before, so it was unknown if sufficient numbers of subjects could be identified or if oral estradiol would be tolerated.

2) What are the key feasibility findings?

A low rate of dual x-ray absorptiometry (DXA) screening at our local center and comorbid conditions potentially increasing the risk for subjects for an adverse event in the intervention group of estradiol precluded investigators from achieving target recruitment numbers. Our center’s adherence to national guidelines for screening for CF-related bone disease with DXA was on par with national averages, but was still low highlighting the need for increased attention to this important extra-pulmonary manifestation of CF. Unfortunately, 21% of patients screened had not had any DXA performed for routine screening in adults with CF. Per national guidelines, patients with low BMD (*T* or *Z* score < − 1) are recommended to have an evaluation for hypogonadism, but none of the patients with low bone mineral density had laboratory evaluation for hypogonadism. The use of systemic estrogen (mostly ethinyl estradiol in contraceptive products) at our center was 16% which was consistent with previously reported rates of 17–30%; however, this was higher than anticipated by physicians in the CF center.

3) What are the implications of the feasibility findings for the design of the main study?

Refinements in future study design may include a wash-out period for women already on estrogen and conducting a DXA test at the time of enrollment. Systematic quality improvement initiatives may also be needed at centers to improve adherence to screening for CF-related bone disease to identify local barriers to having the DXA performed at the recommended intervals. Involvement of an endocrinologist or other expert in metabolic bone disease for interpretation and management of bone disease may also improve screening for CF-related bone disease.

## Introduction

CF is a life-shortening genetic condition affecting approximately 30,000 people in the USA [1]. Previously considered a pediatric disease, recent advancements in therapies for CF have increased the median age of survival above 40 years [1]. This longevity results in an increased prevalence of complications including CF-related bone disease (CFBD), which affects over 26% of adults with CF [1]. CFBD increases the risk for low-impact [2] and vertebral fractures [3]. Thoracic fractures limit patients’ ability to perform daily therapies necessary to maintain optimal lung health [3]. CFBD is a multi-factorial disease stemming from nutritional deficiencies including vitamin D deficiency due to exocrine pancreas insufficiency, chronic inflammation, glucocorticoid therapy, hypogonadism, physical inactivity [3, 4], and the presence of the disease-causing cystic fibrosis transmembrane conductance regulator (CFTR) mutation expressed on osteoblasts and osteoclasts [5].

Although the CF Foundation recommends treatment of hypogonadism in patients with CF and low BMD [3, 6], no clinical trial has evaluated if the bone health of hypogonadal women with CF can be improved by estradiol replacement. Estrogen deficiency increases bone resorption resulting in decreased BMD and increased fracture risk which can be restored with estrogen replacement [7]. The International Osteoporosis Foundation and the International Federation of Clinical Chemistry and Laboratory Medicine recommend monitoring C-terminal telopeptide of collagen I (CTX-1, reflecting bone resorption) and procollagen type I N-terminal propeptide (P1NP, reflecting bone formation) as markers of bone turnover [8, 9]. Markers of bone resorption have been previously described as disproportionately elevated compared to markers of bone formation in patients with CF consistent with inadequate accrual and net loss of bone [10].

Women with CF have pubertal delay, irregular menses, fertility problems, and decreased quality of life [11], which can all be related to untreated hypogonadism. Oral contraceptives and condoms are the most common forms of contraception used by patients with CF [12]. Retrospective and prospective studies of estrogen supplementation in women with CF have not demonstrated increased morbidity [13–15].

The overall objective of this pilot study was to investigate the feasibility of conducting a future study of the effect of estrogen supplementation on clinical outcomes, bone health, sexual health, reproductive health, lung health, and inflammation in women with CF using a single center, randomized, placebo-controlled, double-blinded trial in women with CF who have low estrogen levels and low BMD.

## Materials and methods

### Overview of study design

**Fig. 1:**
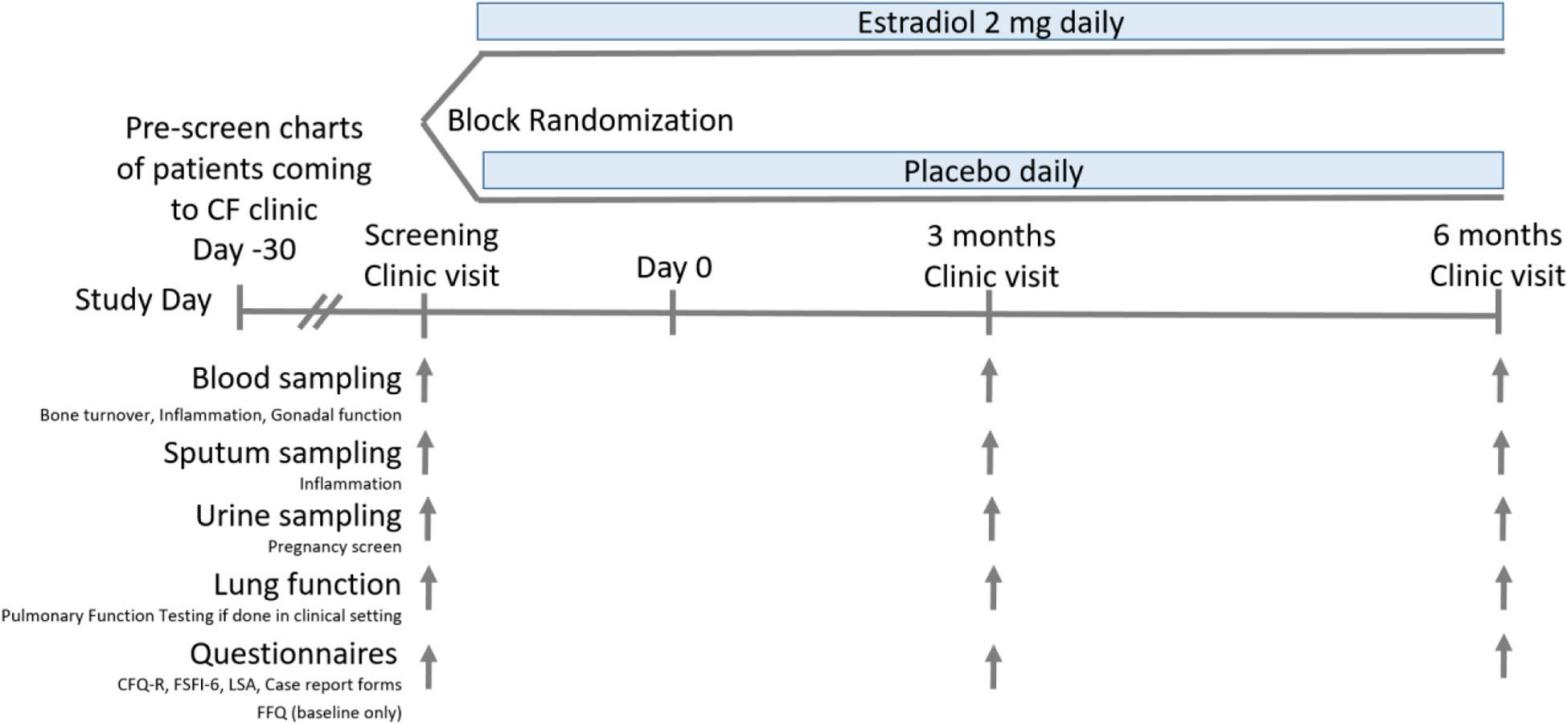
Overview of study design

The overview of the study design is shown in Fig. 1.

#### Hypothesis and aims

We hypothesized that estrogen supplementation given to hypogonadal women with CF and low BMD would improve their bone health. As a feasibility study, the primary aim was to test feasibility metrics of recruitment, retention, adherence, and completion of participants. The secondary aim was to test whether 2 mg of oral estradiol taken daily for 6 months would raise a subjects’ serum estradiol concentration compared to placebo. Additional exploratory aims were to test whether estradiol supplementation improves bone health as measured by relative changes in C-terminal telopeptide of collagen I (CTX-1) and procollagen type I N-terminal propeptide (P1NP), improves lung function as measured by forced expiratory volume in 1 second (FEV1), reduces inflammation as measured by number and phenotype of polymorphonuclear neutrophils (PMNs) in blood and airway fluid, levels of IL8 and CRP in blood and level of IL8, IL1beta, and neutrophil elastase in airway fluid, and improved quality of life as measured by responses to a disease-specific quality of life survey.

#### Sponsors

This was an investigator-initiated study supported by departmental funds from the Emory University School of Medicine Department of Medicine.

#### Subjects

Study investigators identified potential subjects for the study by reviewing medical charts of patients scheduled for routine clinical care at the outpatient CF center up to one week in advance of the scheduled clinic visit. Patients were assessed for eligibility based on the inclusion and exclusion criteria by examining the electronic medical record and interviewing the subject during their clinic visit. Potential study participants were approached for informed consent. Patients whose responses suggested that they were likely hypogonadal would be considered to meet the inclusion criteria of hypoestrogenism, and they would be offered the opportunity to participate in the study so that subjects would not have to return for a baseline study visit to collect baseline data. Subjects’ baseline estradiol levels would be confirmed to be less than 91.8 pmol/L before the subject would be randomized.

The following criteria were used to determine eligibility for participation:

##### Inclusion criteria

(1) Adult and adolescent female CF patients (age between 18 and 50 years), (2) presenting to the CF clinic for routine follow up of CF, (3) hypogonadal women defined as estradiol level < 91.8 pmol/L, (4) DXA within 2 years of enrollment with *T* or *Z* score < − 1, and (5) able to tolerate oral medications.

##### Exclusion criteria

(1) Inability to obtain or declined informed consent from the subject and/or legally authorized representative, (2) pregnancy, (3) too ill to participate in study based on investigator’s or study team’s opinion, (4) current use of systemic estrogen, (5) history of thromboembolic event within the previous 2 years, (6) history of migraines with aura, (7) hypercoagulability including previous diagnosis of Factor V Leiden or Protein C or S deficiency, (8) current smoker, (9) history of diagnosis with breast or uterine cancer, (10) current significant liver disease with cholelithiasis or cirrhosis, (11) status post lung or liver transplantation, and (12) current use of systemic steroids.

#### Intervention: oral estradiol

Subjects would be block randomized in blocks of 4 with a 1:1 allocation ratio to parallel groups of intervention and placebo. The randomization code was performed by a biostatistician not involved in the recruitment of subjects. The study drug would be dispensed by the IDS to the investigators with new labels to keep the study medication blinded to all study investigators, study staff, subjects, care providers, and pharmacists. The intervention group was to receive 2 mg estradiol oral daily for 6 months. The placebo group was to receive lactose. The estradiol and placebo were crushed and placed into capsules that were identical in color, shape and size. The investigational drug service (IDS) would dispense the study drug in two 3-month aliquots. Subject adherence was planned to be assessed by counting the number of pills remaining in the bottle in which the study drug was dispensed.

#### Feasibility metrics

The primary aim of this study was feasibility metrics. Recruitment metrics to be monitored included a number of clinic visits screened per month of recruitment, number of subjects enrolled per month, ratio of enrolled and screen fails. Retention metrics included the number of participants who dropped out and reason for drop out. Adherence metrics included the number of participants who adhered to the assignment and duration of adherence to assigned treatment by participant. Completion metrics included the number of participants who completed all procedures and duration from initiation of recruitment to completion of last study procedure by last participant.

#### Procedures

Approximately 16 mL of blood would be collected from subjects’ peripheral vein into Benton-Dickinson vacutainer tubes (Franklin Lakes, NJ): one 4 mL serum separator tube (SST) and two 6 mL K_2_EDTA tubes. The EDTA tubes were to be prepared as previously described [16] by sequential centrifugation at 400G to generate a cell pellet, and then the platelet-rich supernatant was to be centrifuged at 3000G to isolate platelet-free plasma for analysis. The cell pellet was to be resuspended with sterile PBS and analyzed by flow cytometry. The platelet-free plasma and serum would be stored at − 80 °C until batch analysis of biomarkers of inflammation and bone turnover could be performed.

Estradiol and bone turnover markers were to be measured from stored serum. Estradiol concentration was to be determined at baseline, 3-month and 6-month study visits by competitive enzyme immunoassay (Parametric Estradiol Kit, Catalog number KGE014, R&D Systems, Minneapolis, MN). CTX-1, reflecting bone resorption, and P1NP, reflecting bone formation, were to be measured at all study visits by automated chemiluminescent assay (Catalog numbers: IS-3000 and IS-4000, Immunodiagnostic Systems, Gaithersburg, MD). As changes in bone mineral density were not anticipated after only six months of intervention, DXA was not planned to be a measured outcome in this feasibility study.

Markers of inflammation: IL8 (Quantikine ELISA Human IL8/CXCL8 Immunoassay, Catalog number D8000C, R&D Systems, Minneapolis, MN) and C-reactive protein (Quantikine ELISA Human CRP Immunoassay, Catalog number DCRP00, R&D Systems, Minneapolis, MN) were to be measured at all study visits from plasma.

Sputum was to be collected from the study subjects. Sputum specimens were to be prepared as previously described [16]. Expectorated sputum was to be mixed with ice-cold PBS, then mechanically dissociated via slow passage through a needle and then centrifuged at 400G to isolate cell pellet, and then the supernatant was to be centrifuged at 3000G to isolate airway fluid for analysis. The sputum cell pellet was to be resuspended in PBS and analyzed by flow cytometry. The airway fluid would be stored at − 80 °C until batch analysis of biomarkers of inflammation could be performed.

Markers of inflammation: IL8, IL1beta (Quantikine ELISA Human IL-1β/IL-1F2 Immunoassay, Catalog number DLB50, R&D Systems, Minneapolis, MN), and neutrophil elastase (Human Neutrophil Elastase, Catalog number DY9167-05, R&D Systems, Minneapolis, MN) were planned to be measured at all study visits from airway supernatant.

After processing, the cell pellets from expectorated sputum and plasma specimens were to be assessed immediately for PMN count and phenotype by flow cytometry. Surface antibody markers of PMNs (CD45, CD63, CD66b, human neutrophil elastase (hNE), and arginase 1 (ARG1)) were to be used to identify PMNs to be enumerated and characterize their phenotype. CD45 is a marker of leukocytes; granulocytes including neutrophils can be differentiated from other leukocytes by their scatter and other antibody markers including CD115 which would identify monocytes and macrophages to be excluded from PMN counts. A live/dead stain would be used to exclude dead PMNs from the PMN counts. Raw flow cytometry data would be analyzed using FloJo software and compared using non-parametric statistics available in JMP Pro 13.0.

Subjects were scheduled to provide voided urine specimen for assessment of pregnancy status prior to enrollment by chromatographic immunoassay in individualized urine cassettes (AimStep pregnancy test urine cassette tests, Germaine Laboratories, San Antonio, Texas).

Three questionnaires were planned at each study visit. (1) Cystic Fibrosis-Questionnaire-Revised (CFQ-R): a disease-specific quality of life questionnaire and patient-reported outcome. (2) The Life Space Assessment: a patient mobility questionnaire validated for CF; it has been shown to correlate with physical activity in adults with stable CF [17] and pulmonary function [18]. (3) Female sexual function index-6: a brief tool to evaluate female sexual dysfunction. At baseline, a food frequency questionnaire would also be administered to estimate calcium and vitamin D intake.

#### Statistical analysis plan

Although this was a pilot study, we still performed a sample size calculation based on the efficacy of the proposed intervention. We calculated a sample size of 21 participants in each arm to be sufficient to detect a 2-fold increase in estradiol concentration comparing treatment and placebo groups based on a previous study in post-pubertal young adult women with CF [19]. We anticipated a 20% loss to follow-up and therefore proposed to recruit 26 subjects per arm.

Clinical and demographic factors, treatment details, and study outcomes would be described using standard statistical methods. Changes in study outcomes between groups were to be compared using the Student’s *t* test and using two-way repeated measures ANOVA assessing for differences by time, by group (estradiol vs placebo), and the group-by-time interaction.

#### Ethics and data safety

This study was approved by the Emory University Institutional Review Board (IRB) (IRB00107135). It was registered at clinicaltrials.gov (NCT03724955). During study visits, PI and co-investigators would monitor for adverse events including nausea, abdominal cramping, bloating, breast tenderness, thrombotic event, gallstones requiring surgery, and acute pulmonary exacerbations of CF.

#### Confidentiality

All paper records and case report forms were planned to be kept in locked file cabinets prior to data entry into the electronic database. Each subject was to be labeled with a unique study identifier which would be used to link identifiable health information to their de-identified information. Electronic files containing restricted or confidential information were protected by password and encryption.

## Feasibility and preliminary results

**Fig. 2:**
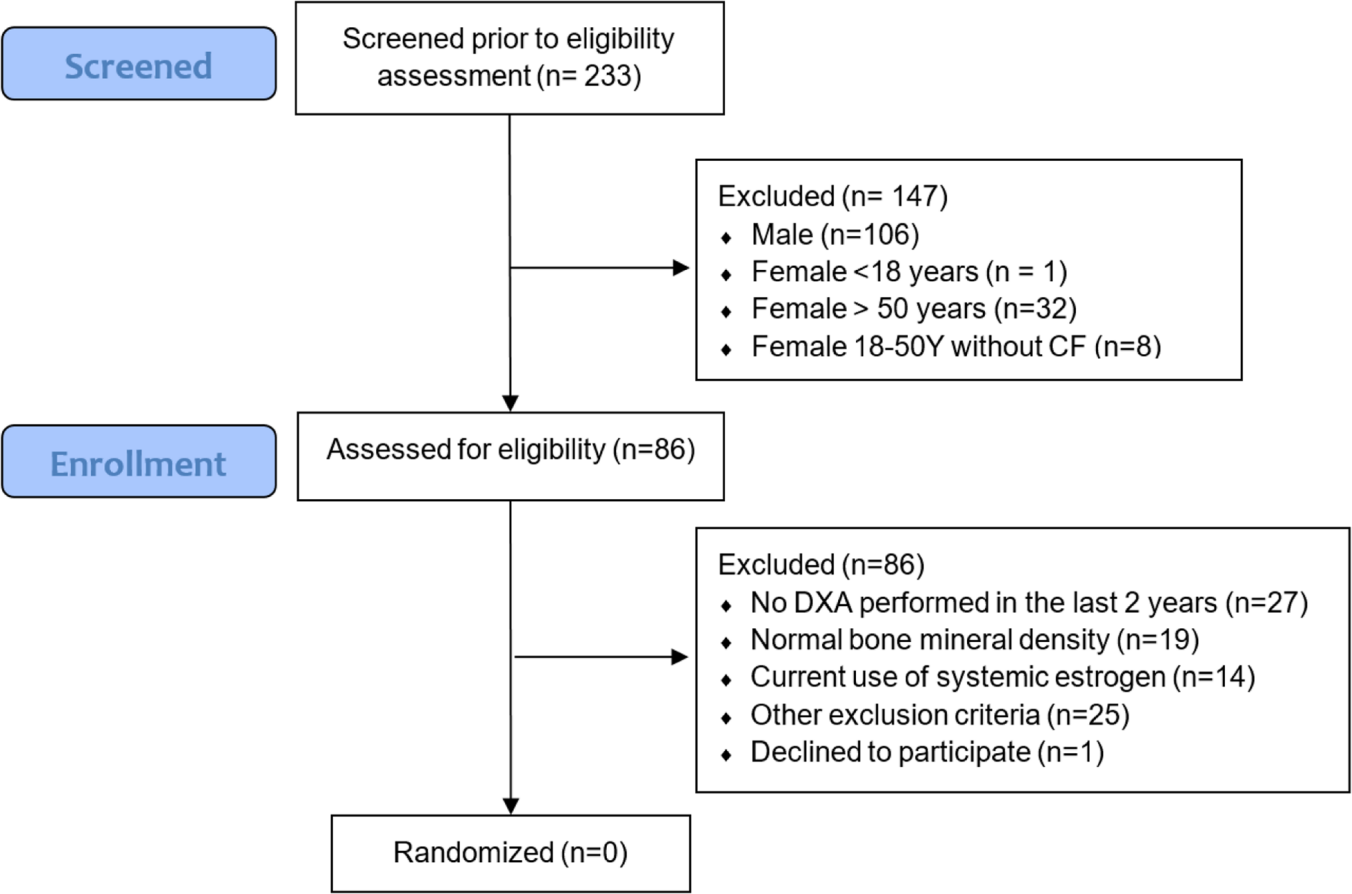
Consort flow diagram

The study was initiated on January 24, 2019. Subjects were screened for enrollment in the study as described in the sections above. After 12 weeks, we had screened 351 clinic visits and found only 1 subject who was eligible based on the study protocol design (Table 1). A review of all CF center clinic visits was conducted to ensure that no possibly eligible subjects had been missed during the screening period. The reasons for subjects’ ineligibility for the trial are presented in Tables 2 and 3. If a patient had already been screened but was scheduled for another CF center visit during the screening period, the patient was screened again. A total of 86 unique women with CF between the ages of 18 and 50 years were screened for eligibility (Fig. 2). Their baseline characteristics are presented in (Table 4). The most common reason for a screen fail was not having had a DXA performed (24%). Given the high rate of ineligibility for the trial, the study was terminated on May 3, 2019.

**Table 1:** Feasibility metrics averaged per month of recruitment

Number of subjects screened for eligibility per month	117
Number of female subjects with CF aged 18–50 years screened for eligibility per month	41.3
Number of eligible screens	1
Number enrolled per month	0

**Table 2:** Summary of findings of screening clinic visits

Total clinic visits screened	351
Exclusion: Male sex	154
Exclusion: Female patient 18–50 years screened but did not come to appointment	23
Exclusion: Female sex, > 50 years	11
Exclusion: Female sex, < 18 years	1
Exclusion: Female sex, does not have CF	34
Clinic visits reviewed of women with CF 18–50 years	128

**Table 3:** Reasons screened patients were ineligible

Women with CF between 18 and 50 years seen in CF clinic	86
Exclusion: pregnancy	3
Exclusion: current use of systemic estrogen	14
Exclusion: thromboembolic event in the previous 2 years	2
Exclusion: significant liver disease with cholelithiasis or cirrhosis	1
Exclusion: current smoker	2
Exclusion: current use of systemic steroids	2
Exclusion: status post lung transplant	2
Exclusion: DXA *T* or *Z* score > -1	19
Exclusion: no DXA result documented	21
Exclusion: no DXA performed in the last 2–5 years	3
Exclusion: no DXA performed in the last 2 years	3
Exclusion: too ill to participate	13
Exclusion: declined informed consent	1

**Table 4:** Baseline characteristics of screened subjects

Characteristic	Mean (SD) or %
Mean age (years)	29.47 (7.63)
Female	100%
Caucasian	83.7%
F508del homozygous	47.7%
At least one copy of F508del *CFTR* mutation	83.7%
Use of CFTR modulator	44.2%
Average BMI (kg/m^2^)	22.6 (5.0)
FEV1 (% predicted)	66.0 (26.1)
CF-related diabetes	31.4%
Exocrine pancreatic insufficiency	89.5%
Should have been screened for hypogonadism due to having low BMD	23.2%

## Conclusions

Estrogen therapy for women is a widely available, relatively well-tolerated, and relatively inexpensive therapy compared to anabolic and anti-resorptive agents to promote bone health. Our feasibility study was designed to provide preliminary evidence for a trial that would generate data to support or refute the current international recommendations that hypogonadal women with a low BMD (*T* or *Z* score < − 1) receive estrogen therapy for the treatment of their CFBD [2, 3].

Our study’s inclusion criteria were designed to identify these women for whom estrogen would be recommended according to the current guidelines. The exclusion criteria were selected to exclude women who would be at higher risk for complications of estrogen therapy such as thromboembolic event and exclude women with confounders affecting bone health (use of systemic steroids or having had a transplant). The strict inclusion and exclusion criteria resulted in the exclusion of nearly all potential subjects who were screened.

If we were to re-attempt to test the hypothesis that estrogen supplementation for hypogonadal women with CF improves BMD as measured by DXA, then we would not include a requirement that patients have a low BMD to be eligible because hypogonadal women are expected to have bone loss regardless of their initial starting BMD. Another strategy would be to incorporate a DXA measurement as part of the study design. Similarly, in redesigning the study, we would include women older than 50 years of age. These older women were excluded as presumptively postmenopausal which is a hypogonadal state. A brief, 6-month, delay in the standard of care of estrogen therapy is brief in the context of 10 years of recommended estrogen replacement after menopause onset. Few women screened were over 50 years of age, likely reflecting that although the median age of survival is now over 40 years, fewer patients with CF are over 50 years old among the population followed at a CF center. Including women over 50 years may not have significantly increased the pool of potentially eligible subjects. While the overall rate of osteoporosis screening with DXA was below the national recommendations, the rate of screening at our CF center for CFBD with a DXA was on par with national averages [1].

The prevalence of estrogen use in the population was informally estimated by physicians in the CF center to be around 10%. During the screening process, the use of estrogen was found to be 16% which is similar to previously reported rates of 17–30% contraceptive use by women with CF at two adult CF centers and Poland [20–22]. None of the women screened that were taking estrogen were prescribed estrogen by providers affiliated with the CF center, and so women who may have been interested in taking estrogen for whatever reason such as regulation of menses or contraception could already have sought estrogen prescription from other healthcare providers outside the CF center such as primary care physicians or gynecologists and the remaining patients not taking estrogen might not be interested in participating in a study where the intervention was estrogen. The average dose of estrogen in oral contraceptives may not be sufficient for restoration of bone density.

In summary, our double-blind, placebo-controlled, randomized feasibility trial in women with cystic fibrosis and estrogen deficiency to test the hypothesis that estrogen improves bone health compared to placebo failed to reach its enrollment targets. The failure to recruit for this study was primarily due to study protocol exclusions including women with ages out of range of the designed protocol, current use of estrogen therapy, bone density results out of range or not available, or too ill to participate. A randomized prospective study is still needed to provide evidence to support the current international recommendations to treat women with cystic fibrosis with low bone mineral density and hypogonadism with sex steroid replacement. Refinements in future study design may include a wash-out period for women already on estrogen, conducting a DXA test at the time of enrollment, and/or extending the inclusion/exclusion criteria to women of a wider range of ages and estrogen status.

**Table:** 

Lessons Learned about Recruitment: Low rates of routine clinical screening for CF-related bone disease by DXA limited the number of patients who could be evaluated for potential participation in this feasibility study. Design of future trial should include a baseline DXA to avoid relying on data collected for routine clinical care. More attention is needed on CF-related bone disease as national screening rates for CF-related bone disease are low, with a median of 54.2% of individuals with CF followed at a CF center who have had a DXA in the last 5 years. Current national recommendations are that all adults with CF have a DXA at least every 5 years, and more often if the DXA *T* or *Z* score is <-1. The intervention in this feasibility study, oral estradiol, should not be used in conjunction with ethinyl estradiol which is commonly used in contraceptive products. The potential study population should be assessed for baseline use of non-estradiol estrogen products and their potential interest in stopping non-estradiol estrogen products in order to use estradiol. Patients with rare disease, such as cystic fibrosis, may have multiple opportunities to participate in research studies which may prevent their participation in another research study. Coordination of “competing” trials requires oversight and collaboration by investigators and could potentially be overseen by the research arm of the local CF center.	

## Data Availability

The datasets generated and/or analyzed during the current study are not publicly available due confidentiality of subject information but are available from the corresponding author on reasonable request.

## Abbreviations

BMD: Bone mineral density
CF: Cystic fibrosis
CFBD: Cystic fibrosis-related bone disease
CTX-1: C-terminal telopeptide of collagen I
DXA: Dual x-ray absorptiometry
FEV1: Forced expiratory volume in 1 second
P1NP: Procollagen type I N-terminal propeptide
PMNs: Polymorphonuclear neutrophils

## References

[1] Cystic Fibrosis Foundation Patient Registry (2017). Annual Data Report.

[2] Sermet-Gaudelus I, Bianchi ML, Garabedian M, Aris RM, Morton A, Hardin DS (2011). European cystic fibrosis bone mineralisation guidelines. J Cyst Fibros.

[3] Aris RM, Merkel PA, Bachrach LK, Borowitz DS, Boyle MP, Elkin SL, Guise TA, Hardin DS, Haworth CS, Holick MF, Joseph PM, O’Brien K, Tullis E, Watts NB, White TB (2005). Guide to bone health and disease in cystic fibrosis. J Clin Endocrinol Metab.

[4] Anabtawi A, Le T, Putman M, Tangpricha V, Bianchi ML (2019). Cystic fibrosis bone disease: Pathophysiology, assessment and prognostic implications. J Cyst Fibros.

[5] Shead EF, Haworth CS, Condliffe AM, McKeon DJ, Scott MA, Compston JE (2007). Cystic fibrosis transmembrane conductance regulator (CFTR) is expressed in human bone. Thorax..

[6] Putman MS, Anabtawi A, Le T, Tangpricha V, Sermet-Gaudelus I (2019). Cystic fibrosis bone disease treatment: Current knowledge and future directions. J Cyst Fibros.

[7] Cauley JA, Danielson ME, Greendale GA, Finkelstein JS, Chang YF, Lo JC (2012). Bone resorption and fracture across the menopausal transition: the Study of Women's Health Across the Nation. Menopause.

[8] Vasikaran S, Eastell R, Bruyere O, Foldes AJ, Garnero P, Griesmacher A (2011). Markers of bone turnover for the prediction of fracture risk and monitoring of osteoporosis treatment: a need for international reference standards. Osteoporos Int.

[9] Szulc P, Naylor K, Hoyle NR, Eastell R, Leary ET, for the National Bone Health Alliance Bone Turnover Marker P (2017). Use of CTX-I and PINP as bone turnover markers: National Bone Health Alliance recommendations to standardize sample handling and patient preparation to reduce pre-analytical variability. Osteoporos Int.

[10] Ionescu AA, Nixon LS, Evans WD, Stone MD, Lewis-Jenkins V, Chatham K (2000). Bone density, body composition, and inflammatory status in cystic fibrosis. Am J Respir Crit Care Med.

[11] Kazmerski TM, Sawicki GS, Miller E, Jones KA, Abebe KZ, Tuchman LK, Ladores S, Rubenstein RC, Sagel SD, Weiner DJ, Pilewski JM, Orenstein DM, Borrero S (2018). Sexual and reproductive health behaviors and experiences reported by young women with cystic fibrosis. J Cyst Fibros.

[12] Roe AH, Traxler S, Schreiber CA (2016). Contraception in women with cystic fibrosis: a systematic review of the literature. Contraception..

[13] Kernan NG, Alton EW, Cullinan P, Griesenbach U, Bilton D (2013). Oral contraceptives do not appear to affect cystic fibrosis disease severity. Eur Respir J.

[14] Chotirmall SH, Smith SG, Gunaratnam C, Cosgrove S, Dimitrov BD, O'Neill SJ, Harvey BJ, Greene CM, McElvaney NG (2012). Effect of estrogen on pseudomonas mucoidy and exacerbations in cystic fibrosis. N Engl J Med.

[15] Fitzpatrick SB, Stokes DC, Rosenstein BJ, Terry P, Hubbard VS (1984). Use of Oral Contraceptives in Women with Cystic Fibrosis. Chest..

[16] Tirouvanziam R, Gernez Y, Conrad CK, Moss RB, Schrijver I, Dunn CE, Davies ZA, Herzenberg LA, Herzenberg LA (2008). Profound functional and signaling changes in viable inflammatory neutrophils homing to cystic fibrosis airways. Proc Natl Acad Sci U S A.

[17] Thobani A, Alvarez JA, Blair S, Jackson K, Gottlieb ER, Walker S (2015). Higher Mobility Scores in Patients with Cystic Fibrosis Are Associated with Better Lung Function. Pulmonary Med.

[18] Gottlieb ER, Smith EC, Wolfenden LL, Allman RM, Tangpricha V (2011). Life-space mobility is associated with frequency of hospitalization in adults with cystic fibrosis. Clin Respir J.

[19] Reiter EO, Stern RC, Root AW (1981). The reproductive endocrine system in cystic fibrosis: I. basal gonadotropin and sex steroid levels. Am J Dis Child.

[20] Rousset Jablonski C, Reynaud Q, Perceval M, Nove-Josserand R, Durupt S, Lega JC, Durieu I (2015). Contraceptive practices and cervical screening in women with cystic fibrosis. Hum Reprod.

[21] Korzeniewska A, Grzelewski T, Jerzynska J, Majak P, Soloniewicz A, Stelmach W (2009). Sexual and reproductive health knowledge in cystic fibrosis female patients and their parents. J Sex Med.

[22] Plant BJ, Goss CH, Tonelli MR, McDonald G, Black RA, Aitken ML. Contraceptive practices in women with cystic fibrosis. J Cyst Fibros. 2008;7(5):412–4. 10.1016/j.jcf.2008.03.001. 10.1016/j.jcf.2008.03.001 18387346

